# The Beneficial Effects of Berberine on Vascular Dysfunction in Type 2 Diabetes Are Enhanced by HSP70 Inhibition

**DOI:** 10.3390/biom16070959

**Published:** 2026-06-29

**Authors:** Valentina Ochoa Mendoza, Swasti Rastogi, Conner Weaver, Micheline Rosa Silveira, Kenia Pedrosa Nunes

**Affiliations:** 1 Laboratory of Vascular Biology, Department of Biomedical Engineering and Science, Florida Institute of Technology, Melbourne, FL 32901, USA; vochoamendoz2019@my.fit.edu (V.O.M.);; 2 Department of Social Pharmacy, School of Pharmacy, Federal University of Minas Gerais, Belo Horizonte 31270-901, MG, Brazil

**Keywords:** berberine, HSP70, diabetes, vascular dysfunction, hypercontractility

## Abstract

Type 2 diabetes (T2D) is a chronic metabolic disorder leading to increased cardiovascular risk and vascular dysfunction. Hyperglycemia, a hallmark of T2D, drives hypercontractility, thereby compromising vascular function. Heat shock protein 70 (HSP70) has emerged as an important player in vascular reactivity under physiological conditions via its interaction with calcium mobilization, and in T2D, blocking this protein prevents hypercontractility. Circulating extracellular HSP70 (eHSP70) has also been proposed as a biomarker in chronic diseases, as it can function as a damage-associated molecular pattern (DAMP) to activate the innate immune system and promote low-grade inflammation. Berberine (BBR), a natural alkaloid with anti-inflammatory properties, has been shown to attenuate vascular contraction by modulating intracellular calcium handling. Yet the link between HSP70 and BBR in modulating vascular contraction in T2D remains unknown. Therefore, we investigated whether acute and/or chronic BBR treatment modulates HSP70 to prevent vascular hypercontractility in the T2D mouse model. For acute ex vivo treatment, db/+ and db/db aortic rings were incubated for 30 min with or without the HSP70 inhibitor VER155008, in the presence or absence of BBR or vehicle. For chronic in vivo treatment, db/+ and db/db mice received intraperitoneal BBR injections (10 mg/kg, 3 times per week) and BBR in their drinking water (0.5 mg/mL) for 28 days. Following chronic (4 weeks, in vivo) or acute ex vivo (30 min) BBR treatment, vascular function was assessed in aortic rings isolated from male T2D (db/db) and age-matched non-diabetic (db/+) mice using wire myography. Rings were incubated with or without the HSP70 inhibitor VER155008, in the presence or absence of BBR or vehicle. Overt hyperglycemia and hypercontractility were observed in diabetic animals compared with non-diabetic controls. While acute BBR treatment attenuated vasoconstriction in both diabetic and nondiabetic groups, the combination of BBR and VER155008 produced a stronger inhibitory effect only in the diabetic group. Chronic BBR treatment prevented aortic hypercontractility in diabetic mice; however, the synergistic effect with VER155008 was no longer observed. Additionally, BBR reduced systemic HSP70 levels. Collectively, these findings indicate that BBR improves vascular smooth muscle cells’ function in T2D, at least in part, through HSP70-dependent mechanisms during chronic treatment.

## 1. Introduction

Diabetes is a prevalent chronic disease projected to affect over 640 million people worldwide by the year 2040 [1,2]. T2D, the predominant form of diabetes, accounts for more than 90% of cases globally and is a major risk factor for cardiovascular disease. The clinical burden of T2D is driven not only by impaired glucose homeostasis but also by progressive vascular complications, which includes endothelial dysfunction and smooth muscle hypercontractility resulting in blood vessel damage [3,4]. This scenario contributes substantially to morbidity, mortality, and healthcare costs [5], underscoring vascular dysfunction as a central feature of T2D pathophysiology.

The development and/or progression of vascular dysfunction in T2D is multifactorial and involves alterations in intracellular calcium (Ca^2+^) signaling. In vascular smooth muscle cells (VSMCs), Ca^2+^ is the primary determinant of contraction and is tightly regulated by influx through voltage-dependent and -independent channels, as well as release from intracellular stores [6,7,8]. Hyperglycemia disrupts these regulatory mechanisms, contributing to vascular dysfunction. BBR, a bioactive compound with established anti-inflammatory properties, has been shown to modulate Ca^2+^ handling and improve vascular function [9,10,11,12]. This natural alkaloid has been used in traditional Asian medicine for centuries and gained increasing attention for the treatment of chronic metabolic diseases [6,9]. In diabetic models, BBR improves insulin resistance, hyperglycemia, and inflammation. Chronic BBR administration reduces cerebral arterial hypercontractility, improves vascular reactivity in diabetic rats, and decreases intracellular Ca^2+^ [13]. In mouse aortic rings, BBR promotes vasorelaxation and attenuates contraction, partly by inhibiting transient receptor potential vanilloid 4 (TRPV4)-mediated Ca^2+^ influx and downstream calcium-calmodulin/myosin light chain (CaM/MLC) signaling [14]. Collectively, these findings indicate an interplay between BBR and Ca^2+^ handling in VSMCs. HSP70, a well-known chaperone, emerged as a key player in keeping smooth muscle functionality as it influences Ca^2+^ mobilization and contractile signaling in VSMCs under physiological and diabetic conditions. More specifically, ex vivo blockade of HSP70 improves vascular function in diabetic aortic rings by decreasing free Ca^2+^ level [15,16]. Furthermore, we and others have shown that circulating extracellular HSP70 (eHSP70) is elevated in chronic diseases such as T2D and may contribute to a pro-inflammatory milieu by acting as a DAMP, by binding to innate immune receptors in the vasculature [17]. In diabetes, higher eHSP70 levels have been associated with disease duration and vascular dysfunction [18]. Still, whether HSP70 mediates the beneficial effects of berberine on vascular contractility in T2D remains unknown. Interestingly, in a mouse model of heat stress, BBR administration prior to heat exposure prevented the heat-induced upregulation of HSP70 and TNF-α, a major proinflammatory cytokine. In the same study, BBR pretreatment also inhibited HSP70 and TNFα expression in PC12 cells in a dose- and time-dependent manner [19]. Based on these observations, the present study aimed to investigate whether BBR treatment abolishes aortic hypercontractility via HSP70. To address this gap, contraction was assessed in aortic rings from T2D db/db mice following acute and chronic BBR treatment, with and without vehicle or VER155008, an HSP70 pharmacological inhibitor. Additionally, given that increased systemic HSP70 is associated with low-grade inflammation in diabetes, and whether BBR could affect it is unknown, serum HSP70 levels were evaluated following chronic BBR treatment.

## 2. Materials and Methods

### 2.1. Ethics Statement

All animal procedures were reviewed and approved by the Institutional Animal Care and Use Committee of the Florida Institute of Technology (IACUC; Protocol #00000001; approved 12 May 2025) and conducted under veterinary supervision in accordance with the ARRIVE guidelines. The principles of Replacement, Reduction, and Refinement (3Rs) guided all aspects of study design to minimize animal use and distress while maintaining scientific rigor. Sample sizes were determined based on statistical considerations to ensure adequate power. All personnel responsible for animal handling and experimental procedures were appropriately trained and certified in accordance with institutional and IACUC standards. Experimental protocols were designed to limit pain and discomfort, with anesthesia and analgesia administered before and after procedures per veterinary recommendations. Humane endpoints were established to safeguard animal welfare throughout the study.

### 2.2. Animals

Diabetic db/db mice (B6.BKS(D)-Leprdb/J; homozygous for Leprdb), and age-matched db/+ heterozygous littermates aged 12 weeks were purchased from The Jackson Laboratory. Mice were maintained on a 12:12 h light-dark cycle and fed a standard chow diet with water ad libitum. Animals were housed individually or in groups of 2–4 per cage with standard enrichment, including nesting material, a shelter, and a gnawing block.

For the chronic BBR treatment experiment, male db/+ and db/db mice were randomly allocated at 16 weeks of age into four groups: db/+ vehicle, db/db vehicle, db/+ berberine, and db/db + berberine, with *n* = 6 mice per group. Berberine doses for the drinking-water, intraperitoneal injections, and ex vivo functional protocols were selected based on prior published studies [20,21,22]. Glucose levels were measured biweekly until the time of sacrifice. After 28 days of treatment, db/db and db/+ mice were weighed, and non-fasting blood glucose was measured using a Metene TD-4116 blood glucose monitor. Mice were then anesthetized with 2.5% isoflurane in 100% O_2_, and blood was collected from the abdominal aorta using a BD Vacutainer serum collection tube. Serum was separated and snap-frozen in liquid nitrogen for subsequent Western blot analysis. Animals were then euthanized by exsanguination via cardiac puncture. The thoracic aorta was excised, immersed in cold physiological salt solution (PSS), cleaned of surrounding adipose tissue, and used for vascular function studies on a wire myograph or snap-frozen for later analysis. The endothelium was mechanically removed by gently rubbing the luminal surface with fine forceps, and successful denudation was confirmed by an acetylcholine viability test.

### 2.3. Ex Vivo Assessment of Thoracic Aortic Function

Thoracic aortic rings (2 mm in length) were isolated, cleared of surrounding connective tissue, and the endothelium was mechanically removed from all experimental animals before mounting on a DMT 620M multi-wire myograph system (Danish Myo Technology, Copenhagen, Denmark) under isometric conditions. Rings were adjusted to an optimal resting tension of approximately 5 mN, as determined in previous studies [16] in PSS, continuously aerated with carbogen (95% O_2_, 5% CO_2_) and maintained at 37 °C. During a 1-h equilibration period, the PSS was replaced every 15 min, and the resting tension was readjusted to 5 mN as needed. Functional integrity of each ring was confirmed by exposure to a high-potassium (KCl) depolarizing solution (120 mM). Rings that developed active tension greater than 50% of the resting preload were considered viable, rinsed with fresh PSS, and allowed to re-equilibrate for approximately 30 min before experimental protocols. For each experimental comparison, rings derived from the same animal were allocated across treatment conditions whenever possible to permit intra-animal comparisons and reduce biological variability.

### 2.4. Acute Treatment with Berberine Chloride

Thoracic aortic rings from male db/+, and db/db mice were incubated in PSS containing BBR (10^−5^ M) (CAS number 633-65-8) purchased from Sigma-Aldrich, diluted in DMSO for 30 min at 37 °C under continuous aeration with a gas mixture of 95% O_2_ and 5% CO_2_. Control rings were incubated with vehicle (DMSO) alone. Four experimental groups were used: (1) db/+ (control), (2) db/+ BBR for 30 min, (3) db/db (diabetic), and (4) db/db + BBR for 30 min.

### 2.5. Chronic Treatment with Berberine Chloride

Diabetic (db/db) and non-diabetic control (db/+) mice received BBR (CAS number 633-65-8) purchased from Sigma-Aldrich (10 mg/kg) via intraperitoneal injection three times per week (Monday, Wednesday, and Friday) for 28 days (12 total injections), and daily BBR (CAS number 633-65-8) purchased from Sigma-Aldrich was added to their drinking water. Control animals received vehicle-only injections on the same schedule. BBR was dissolved in Milli-Q water to create a concentrated stock solution. This stock was diluted further in drinking water to achieve a final concentration of 0.50 mg/mL (approximately 2.5 mg/day per mouse). Daily water intake was monitored to ensure consistent dosing.

### 2.6. Time–Force Curve Analysis

For time–force analysis, thoracic aortic segments were incubated for 30 min with either vehicle (DMSO) or the HSP70 inhibitor VER155008 (10 µM), followed by stimulation with phenylephrine (PE; 10^−6^ M). Isometric tension was recorded continuously for 15 min at a 1-s sampling rate using the DMT data acquisition system. Time–force curves were fitted to a bi-exponential function using the least-squares regression. The phasic component was defined as the peak force corresponding to the fast-to-slow transition, while the tonic component was defined as the sustained tension at 15 min. Both parameters were expressed relative to the baseline tension at time 0.

### 2.7. Serum Collection and ELISA

Blood was collected from the abdominal aorta into BD Vacutainer Serum Blood Collection Tubes and allowed to clot at room temperature for 30 min. Samples were then centrifuged at 3500× *g* for 25 min, and the serum was carefully collected and stored at −80 °C until analysis. Circulating extracellular HSP70 (eHSP70) levels in serum were measured using a commercially available AMP’D High Sensitivity HSP70 ELISA Kit (Enzo Life Sciences, Farmingdale, NY, USA; ENZ-KIT-101) according to the manufacturer’s instructions.

### 2.8. Western Blot

Briefly, 10 μL of serum or 10 μg of tissue from each sample was separated by 10% SDS-PAGE and transferred to nitrocellulose membranes. Membranes were stained with Ponceau S for 30 s, imaged, and rinsed for 5 min in TBST. Non-specific binding was blocked by incubating the membranes for 1 h at room temperature in 5% nonfat dry milk in TBST. Membranes were incubated overnight at 4 °C with a primary anti-HSP70 antibody (Cell Signaling Technology, Danvers, MA, USA, Cat. # 4872; 1:2000 in TBST containing BSA), followed by 1 h incubation at room temperature with an anti-rabbit secondary antibody (Cell Signaling Technology, Danvers, MA, USA, Cat. # 7074; 1:10,000 in TBST) under gentle agitation. Signal was developed using SuperSignal West Femto Substrate (Thermo Fisher Scientific, Rockford, IL, USA, Cat. #34095) and imaged on a ChemiDoc MP Imaging System (Bio-Rad, Hercules, CA, USA). After chemiluminescent imaging, membranes were stained with Revert 700 Total Protein Stain (LI-COR Biosciences, Lincoln, NE, USA, No. 926-110116) for normalization. Briefly, membranes were rinsed in 1× TBST for 5 min, stained for 5 min with freshly prepared Revert 700 solution, and reimaged using the colorimetric setting. Band intensities were quantified using ImageJ software version 1.54p (NIH, Bethesda, MD, USA) and normalized to total protein content.

### 2.9. Statistical Analysis

All data analysis and graph generation were performed using GraphPad Prism 10.3.1 (GraphPad Software, San Diego, CA, USA). Data are presented as mean ± SEM. Data were analyzed in a blinded manner whenever possible, and experimental plans included appropriate controls matched to the experimental groups. Normality was assessed using the D’Agostino–Pearson test, and outliers were identified and excluded using Grubbs’ test when appropriate. Acute treatment experiments were analyzed by one-way ANOVA followed by Sidak’s post hoc test, whereas chronic treatment experiments were analyzed by two-way ANOVA followed by Sidak’s post hoc test. A *p*-value ≤ 0.05 was considered statistically significant. The reported sample size (n) refers to the number of mice per group. All experimental procedures were performed in a blind manner whenever feasible. Mice were randomly allocated into treatment groups at the start of the chronic berberine treatment experiment to prevent allocation bias. Appropriate controls were included for each experimental group.

## 3. Results

### 3.1. Animal Profile

**Figure 1:**
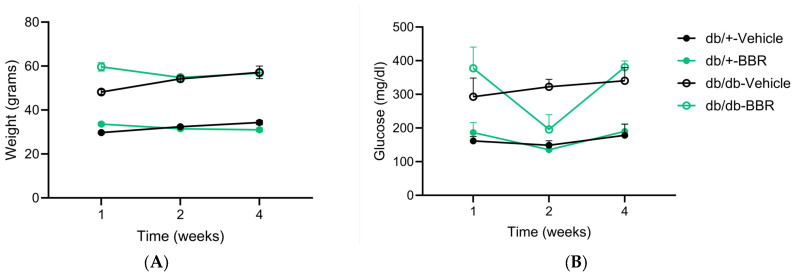
Animal profile following berberine treatment. Panels (**A**,**B**) show fluctuations in body weight (**A**) and blood glucose levels (**B**) over 4 weeks in all groups: non-diabetic (db/+), diabetic (db/db), non-diabetic berberine-treated (db/+BBR), and diabetic berberine-treated (db/db-BBR). Green lines indicate BBR treatment, and black lines indicate non-treated groups. Data represent mean values with error bars indicating standard error of the mean (*n* = 12).

Diabetic (db/db) mice were identified by elevated non-fasting blood glucose levels compared to non-diabetic (db/+) controls (Figure 1). After 4 weeks of treatment, body weight increased in the untreated db/+ controls and untreated db/db mice, while it remained stable in berberine-treated db/db mice. Blood glucose levels increased in both untreated db/+ controls and untreated db/db mice, whereas the berberine-treated db/db group showed no significant change over the treatment period.

### 3.2. Acute Berberine Treatment Attenuates PE-Induced Contraction in Thoracic Aortic Segments with Synergy Observed Only in Diabetic Mice

**Figure 2:**
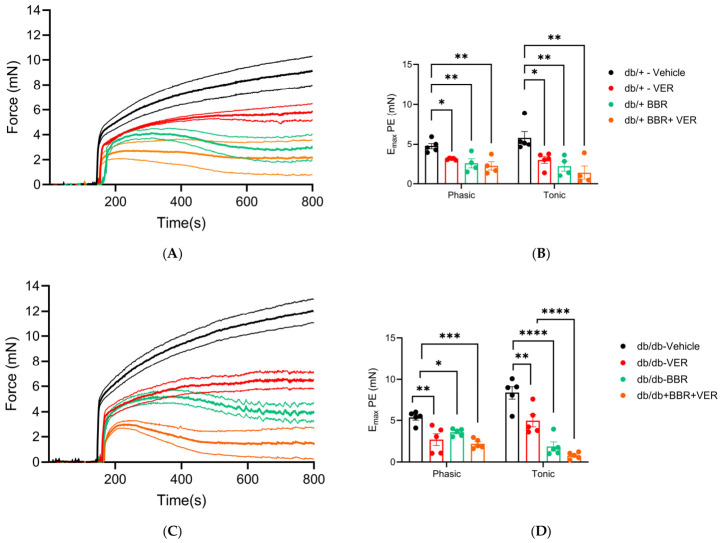
Force development curves show aortic responses to a single dose of phenylephrine (PE) in db/+ (**A**,**B**) and db/db (**C**,**D**) mice. Aortic rings were incubated with vehicle (black), VER155008 (VER; red), berberine (BBR; green), or BBR+VER (orange) for 30 min prior to PE addition. (**A**) Representative single dose curve in the aorta of db/+ mice. (**B**) Emax of phasic and tonic parts of the curve in db/+. (**C**) Representative single dose curve in the aorta of db/db mice. (**D**) Emax of the phasic and tonic parts of the curve in db/db mice. Data represent mean ± SEM; * *p* ≤ 0.05, ** *p* ≤ 0.001, *** *p* ≤ 0.0005, **** *p* ≤ 0.0001 versus corresponding vehicle control (analyzed by one-way ANOVA); *n* = 4–5.

We evaluated the phasic and tonic phases of the biphasic contraction curve, which reflect intracellular and extracellular calcium sources, respectively. Both phases of the curve in db/+ (Figure 2A,B) and db/db (Figure 2C,D) animals were attenuated by treatment with VER, BBR, or VER + BBR compared with the vehicle group. In the db/+ group, all treatments reduced force by ~50% compared with the vehicle, and the combination of BBR+VER did not potentiate the attenuation of force development compared with the BBR group, suggesting that berberine and HSP70 might act via the same mechanism(s) to modulate vascular contraction in the non-diabetic group. Conversely, in the db/db group, the combination of BBR+VER shows synergism, indicating that, in diabetic conditions, their effects on vascular contraction are independent. Additionally, incubation with BBR affected both phases of the contraction curve, suggesting that berberine and HSP70 are involved in calcium mobilization from both internal and extracellular sources during contraction.

### 3.3. Chronic Berberine Treatment Abolishes Hypercontractility in Thoracic Aortic Segments from Diabetic Mice and Is Potentiated by HSP70 Inhibition

**Figure 3:**
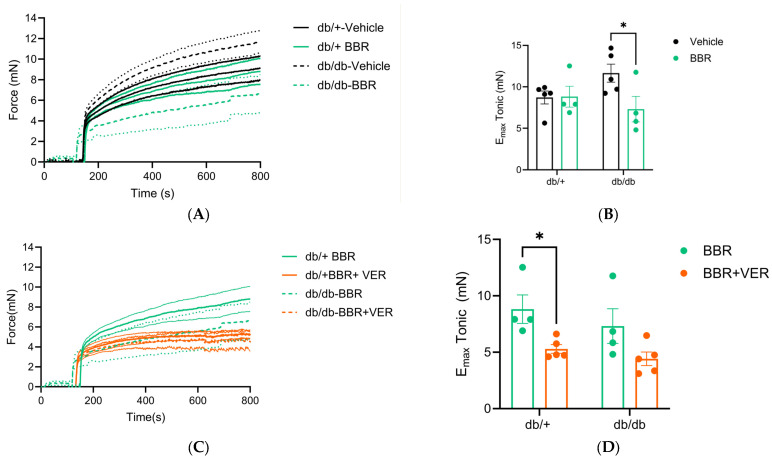
Force development curves to a single dose of phenylephrine (PE). (**A**) Time-force curve of db/+ vs. db/db mice treated with BBR (green) or vehicle (black), (**B**) Maximal tonic force (Emax) for vehicle- and BBR-treated groups. (**C**) Representative time-force of contraction after BBR chronic treatment with (orange) or without VER (green). (**D**) Maximal tonic force (Emax) for BBR- and BBR+VER-treated groups. Data represent mean ± SEM; * *p* ≤ 0.05, were analyzed by two-way ANOVA with Holm–Sidak’s post hoc test; *n* = 4–5.

Aortic contractility was assessed following 4 weeks of treatment with BBR or vehicle in the presence or absence of VER in diabetic (db/db) and non-diabetic (db/+) controls. Chronic treatment with BBR rescued proper vascular contraction in the db/db group by preventing the hypercontractile state in the diabetic group, while no significant differences were observed between the non-diabetic group with vehicle vs. BBR (Figure 3A,B). To evaluate the effects of HSP70 inhibition combined with chronic BBR treatment, aortas from BBR-treated non-diabetic and diabetic mice were preincubated with VER155008 (VER) or vehicle (DMSO) for 30 min prior to stimulation with a single dose of PE. The treated db/db group incubated with VER displayed a ~40% decrease in force development compared to the BBR-treated db/db (Figure 3C,D), showing that HSP70 inhibition enhances the effects of BBR.

### 3.4. Chronic Treatment with BBR Decreases Systemic Levels of HSP70 in T2D Mice

**Figure 4:**
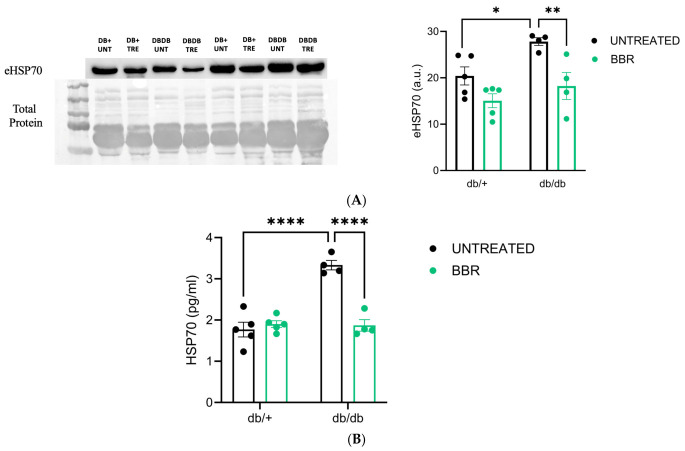
Circulating HSP70 blot (**A**) and ELISA (**B**) of nondiabetic and diabetic mice chronically treated with BBR or vehicle. Data are shown as means ± SEM; * *p* ≤ 0.05, ** *p* ≤ 0.001, **** *p* ≤ 0.0001; *n* = 4–5. Please see Supplementary Materials for the full Western blot image.

Serum analysis confirmed elevated eHSP70 levels in db/db animals compared with non-diabetic db/+ in both Western blot and ELISA assay. There was a significant decrease in circulating HSP70 levels following 4 weeks of treatment with BBR only in diabetic animals, while all other groups (db/+ vs. db/+BBR) showed consistent eHSP70 levels (Figure 4A,B). No difference in eHSP70 was observed between the non-diabetic group receiving BBR treatment and the non-treated group.

## 4. Discussion

HSP70 plays critical roles in all muscle types; deletion of inducible HSP70 genes disrupts Ca^2+^ handling in cardiac and skeletal muscle, and pharmacological inhibition significantly reduces smooth muscle contraction under physiological conditions [15,23,24]. The widespread vascular dysfunction observed in T2D is closely linked to increased cardiovascular risk, as chronic hyperglycemia disrupts multiple signaling pathways, exacerbating vascular complications. Hyperglycemia is associated with elevated circulating eHSP70 levels [18], which can act as a DAMP, activating the immune system and contributing to low-grade inflammation [25]. Studies suggest that strategies targeting HSP70 modulation in T2D may delay the development and/or progression of diabetes [26].

BBR, an isoquinoline alkaloid known to possess a wide range of pharmacological properties, has gained attention as a therapeutic agent due to its antibacterial, antiparasitic, and gastroprotective effects [27]. In addition to its metabolic effects, extensive evidence indicates that BBR provides anti-inflammatory, antioxidant, and cardiovascular benefits in diabetes, protecting against hyperglycemia-induced endothelial dysfunction [11]. Interestingly, in a mouse model of heat stress, BBR administration before heat exposure prevented the heat-induced upregulation of HSP70 and TNFα. In the same study, BBR pretreatment also inhibited HSP70 and TNFα expression in PC12 cells in a dose- and time-dependent manner [19]. Before examining the relationship between BBR and HSP70 in T2D-induced hypercontractility, we conducted pilot studies to determine whether BBR incubation for 24-h could affect HSP70 expression in the mouse aorta. We found that aortic tissues incubated for 24 h with BBR under normal and hyperglycemic conditions presented reduced HSP70 tissue expression (data are included in the Supplementary Materials). Similarly, when incubated over 30 min with BBR in the wire myograph, the force of contraction was reduced to about 50% compared to the control group (See Supplementary Data).

In db/db mice and high-fat-fed rats, BBR has been shown to reduce body weight, improve glucose tolerance and whole-body insulin sensitivity, and lower plasma triglyceride levels without affecting food intake [6]. In agreement with these findings, our diabetic animals treated with BBR for 28 days exhibited reductions in body weight and blood glucose levels during the first two weeks of treatment. However, these effects were not sustained, as both parameters returned to baseline values by the end of the treatment period (Figure 1). While our diabetic animals seemed to have reverted to overt hyperglycemia by the end of treatment, one could argue that the disease progression at the time of treatment was advanced, making BBR’s effect insufficient to fully improve their overall metabolic profile. Yet, significant differences between non-diabetic and diabetic animals persisted.

Here, we show that BBR and pharmacological inhibition of iHSP70 with VER155008 independently reduced vascular contraction in the aortas of both db/+ and db/db mice. In db/+ animals, the combined BBR + VER treatment did not produce a greater effect than either treatment alone (Figure 2A,B), suggesting that BBR and HSP70 may converge on overlapping mechanisms under physiological conditions. In db/db animals, however, the combined treatment produced a synergistic effect (Figure 2C,D) compared with either treatment alone, indicating that under pathological conditions, BBR may engage additional pathways beyond those affected by HSP70 in vasoconstriction. Additionally, while the biphasic contraction curve elicited by PE was attenuated across all diabetic-treated groups relative to the vehicle, this effect appeared to be more pronounced in the latter phase of the response (Figure 2D). This result is consistent with previous work on diabetic animals [16]. Interestingly, studies in rat mesenteric arteries and aorta have shown that BBR inhibits contraction in an endothelium-dependent manner, mediated in part by nitric oxide (NO) [28]. In the present work, the endothelium was mechanically removed because the primary cells of interest were VSMCs. While this limitation excludes endothelial contributions to berberine’s effects, HSP70 assists vascular contraction independent of the endothelium [15].

**Figure 5:**
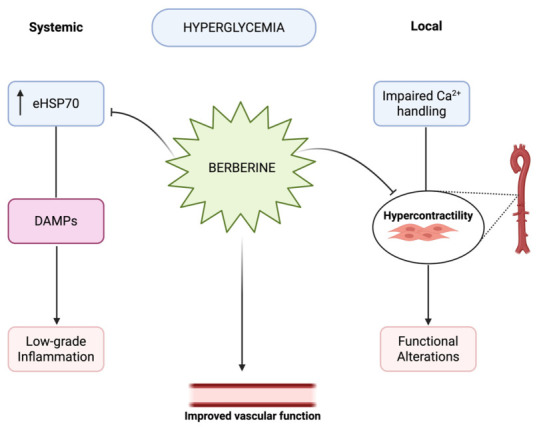
Illustrative figure highlighting the protective effects of BBR on the vasculature of the T2D model. Berberine inhibits two main arms that promote vascular functional alterations in T2D. The former involves the elevated circulating eHSP70 detected systemically, and the latter the hypercontractility due to impaired calcium handling in diabetic aorta. Created with BioRender.com.

Given the effects of BBR on aortic hypercontractility in diabetes following acute exposure in the myograph, we next investigated if chronic treatment would yield similar results and whether that was mediated by HSP70. Our experiments revealed that long-term BBR treatment prevented the hypercontractile phenotype in db/db mice (Figure 3A, B) but did not affect non-diabetic animals. BBR is reported to modulate Ca^2+^ handling through multiple pathways, including those involved in the tonic phase of the biphasic curve [29,30,31,32,33]. In db/db mice, dysregulation of calcium-dependent mechanisms leads to hypercontractility, along with increased expression of pro-inflammatory and pro-fibrotic markers [30]; while BBR and HSP70 are reported to modulate Ca^2+^ handling in the diabetic milieu [13,16], there is no evidence linking BBR to HSP70. To further assess this, we subjected aortic rings from all chronically treated groups to ex vivo incubation with HSP70 inhibitor, VER155008. Our results showed that prolonged BBR exposure promotes HSP70-dependent vascular contraction. Specifically, BBR-treated control and diabetic animals exhibited similar attenuation in maximal force of contraction following VER155008 exposure (Figure 3C,D). Here, one may argue that at the onset of diabetes, HSP70 plays a more prominent role in the diabetic vascular milieu due to its function in stress-related responses. Notably, chronic BBR treatment appears to improve the overall diabetic environment, potentially shifting HSP70 from a direct driver of vascular contraction to a stress marker. Acute HSP70 inhibition suggests that HSP70 helps VSMCs maintain appropriate vascular reactivity, but after several weeks of BBR treatment, one could say that the microenvironment changes so that HSP70’s effects become secondary. Thus, HSP70 likely acts as a modulator of the pathological state rather than the primary mechanism responsible for attenuating vascular contraction. Whether there is a true causal relationship between BBR and HSP70 in the abolition of hypercontractility in diabetes remains to be elucidated. However, to better define BBR’s contribution to the diabetic inflammatory milieu involving HSP70, we next evaluated whether chronic treatment would modulate circulating eHSP70, which is elevated in diabetic patients [18] and thought to promote low-grade inflammation. Serum eHSP70 levels significantly decreased only in diabetic mice receiving chronic BBR treatment compared with untreated diabetic controls (Figure 4A,B). In contrast, no difference in eHSP70 was observed between treated and untreated non-diabetic mice, suggesting that BBR selectively reduces circulating HSP70, which is related to inflammatory signaling under diabetic conditions. In our T2D model, chronic BBR administration selectively lowered eHSP70 levels in diabetic mice, without altering levels in non-diabetic controls, suggesting more relevance in the hyperglycemic milieu (Figure 5).

## 5. Conclusions

In conclusion, our findings support the view that BBR improves vascular function by attenuating aortic hypercontractility in T2D and indicate that these beneficial effects may involve, at least in part, HSP70-related mechanisms. Importantly, chronic berberine treatment was associated with reduced circulating eHSP70 levels in diabetic animals, suggesting that modulation of the systemic HSP70-associated inflammatory milieu might also contribute to the observed improvements in vascular function, although the exact causal relationships remain to be determined. Given that diabetes is associated with vascular dysfunction and that elevated eHSP70 worsens disease progression, our data are clinically relevant and suggest that chronic BBR treatment can target systemic HSP70 and improve vascular function by preventing hypercontractility in diabetes, as illustrated in Figure 5.

## Data Availability

The original contributions presented in this study are included in the article/Supplementary Materials. Further inquiries can be directed to the corresponding author.

## Supplementary Materials

The following supporting information can be downloaded at: https://www.mdpi.com/article/10.3390/biom16070959/s1, Figure S1: schematic representation of experimental protocol for the chronic berberine treatment; Figure S2: Biphasic curve for normoglycemic animals and intracellular heat shock protein 70 expression in aorta; Figure S3: raw Western blot membranes for serum circulating eHSP70 (untreated vs treated db/+, and treated vs untreated db/db).

## Abbreviations

The following abbreviations are used in this manuscript:

T2D	Type 2 Diabetes
BBR	Berberine
iHSP70	Internal HSP70
eHSP70	External HSP70
DAMP	Damage Associated Molecular Pattern
VSMC	Vascular Smooth Muscle Cell
VER	VER155008
DMSO	Dimethyl sulfoxide
