# Supplementary material for: The Beneficial Effects of Berberine on Vascular Dysfunction in Type 2 Diabetes Are Enhanced by HSP70 Inhibition
Source: Biomolecules. 2026 Jun 29;16(7):959. doi: 10.3390/biom16070959 (PMC13406697; doi:10.3390/biom16070959)
Supplement: Supplementary file 1 [file biomolecules-16-00959-s001.zip › biomolecules-4355702-supplementary.pdf]

Figure S1. Schematic representation of experimental protocol for the chronic berberine treatment

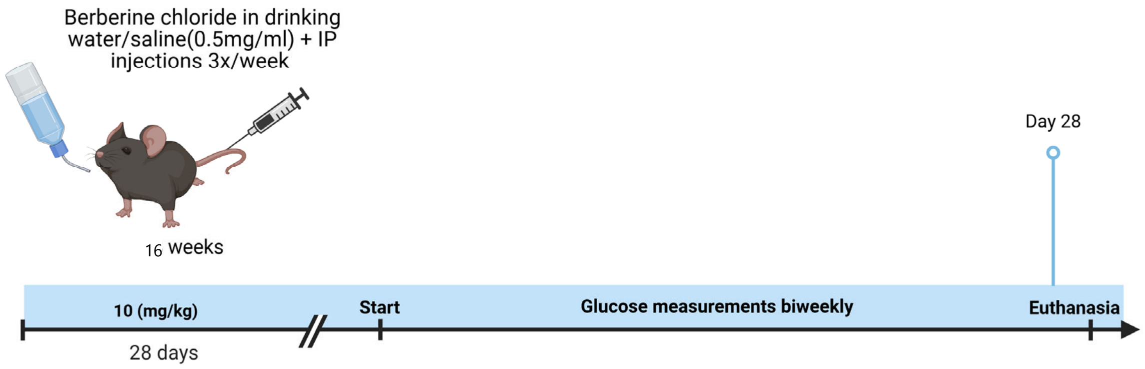

Figure S2. Biphasic curve for normoglycemic animals and intracellular heat shock protein 70 expression

Aortic rings from control C57BL/6 mice were incubated with BBR (10^{-5} M) or vehicle for 30 minutes prior to a single dose of phenylephrine (10^{-6} M). BBR-incubated rings showed a significant attenuation of contraction force compared with the vehicle (DMSO) group (Figure 1.2**A**). While a decrease in the force of contraction tendency was noted on the treated group for the initial part of the curve (phasic force), no significant difference was present between the control and treated groups (Figure 1.2 **B**). Conversely, a decrease of about 50% was reported in the latter part of the curve (tonic phase) in the BBR-treated group compared to its control counterparts (Figure 1.2 **C**). Aortic rings incubated with BBR under normal and high glucose over 24 hours displayed significantly lower levels of HSP70 (Figure 2**D**).

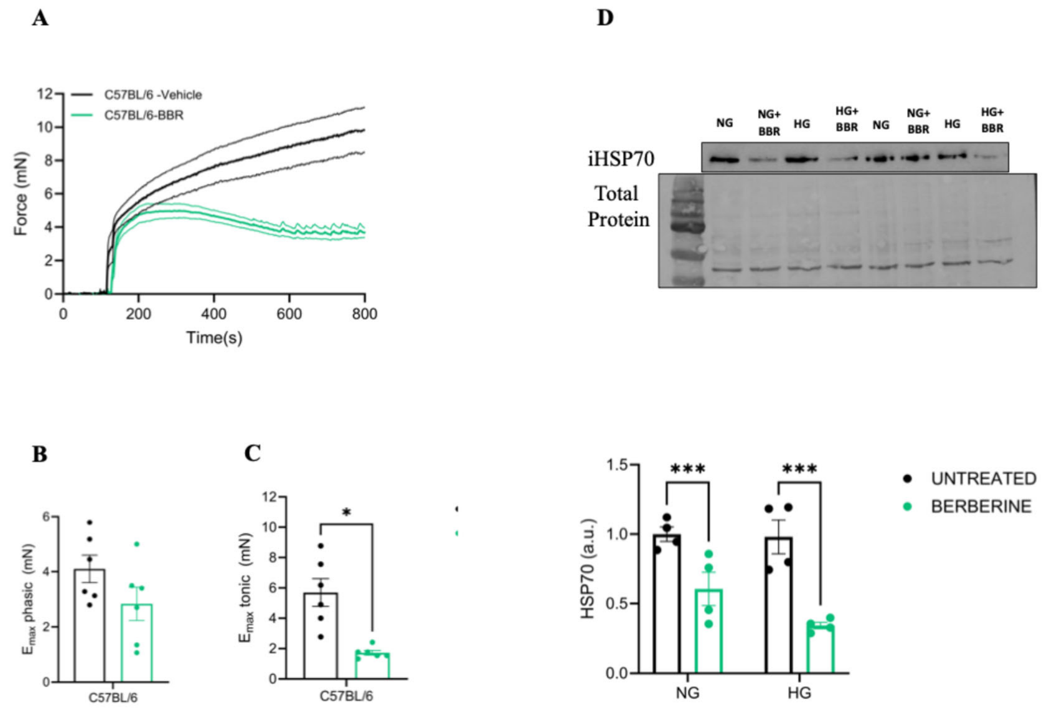

Figure S2: Biphasic curve for normoglycemic animals and intracellular heat shock protein 70 expression. Panel **A** illustrates biphasic force development in aortic rings from C57BL/6 mice, showing a single dose curve to PE in vehicle (black) and BBR (green) aortic rings. Panels **B** and **C** display Emax for the phasic (**B**) and the tonic forces (**C**), respectively, in the control mouse aorta, with vehicle (black) or BBR (green). Panel **D** shows a representative Western blot (iHSP70) and densitometry Data are shown as means ± SEM (n = 4-6 rings per group); $*p < 0.05$ indicates a significant difference versus vehicle control.

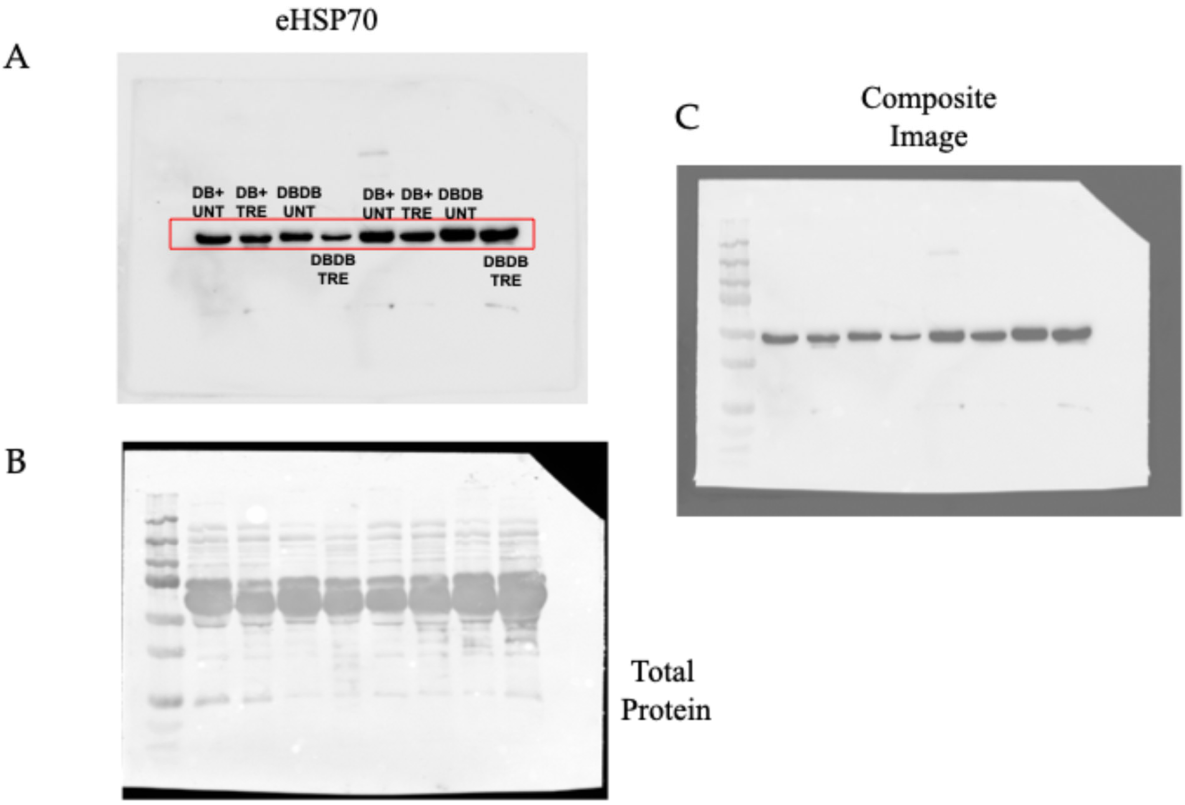

Figure S3 Raw Western blot membranes for serum circulating eHSP70 (untreated vs treated db/+, and treated vs untreated db/db.(**A**) Serum eHSP70 whole blot (treated db/+ and db/db vs untreated db/+ vs db/db). (**B**) Total protein normalization. (**C**) Com posite of the membrane with both the molecular weight and eHSP70
